# University of Maryland, Dental Department Annual Commencement

**Published:** 1883-04

**Authors:** 


### EDITORIAL, ETC.

*University of Maryland Dental Department.*—The Seventy-Sixth Annual Session, Commencement of the University of Maryland, but the first of the Dental Department, was held at the Academy of Music, Baltimore, on the 15th of March, 1883. A very large audience was present, and upon the stage, which was set with an elegant garden scene, were seated the Faculty of the University and a large number of guests, many of them from a distance. The exercises were opened with prayer by Rev. J. S. B. Hodges, S. T. D. of St. Pauls' P. E. Church, after which the mandamus was read, and the dental graduates announced by the Dean of the Dental Department, Prof. F. J. S. Gorgas. The degree of 'Doctor of Dental Surgery' was then conferred upon the following gentlemen, thirty-four in number:

| | |
|---|---|
| J. Hardin Baldwin, | *Kentucky.* |
| Austin F. Banks, | *Michigan.* |
| Bartow B. Breeden | *South Carolina.* |
| Paul Campbell, | *New York.* |
| Frank G. Conklin, | *New York.* |
| Joseph W. Curtis, | *New Jersey.* |
| Erastus S. Dashiell, | *Maryland.* |
| Newton W. Denton, | *Virginia.* |
| R. Delamer Dodson, | *Pennsylvania.* |
| John F. Garrett, | *North Carolina.* |
| Godfrey J. Grempler, | *Maryland.* |
| George W. Hotaling, | *New York.* |
| R. Arthur Hungerford | *Maryland* |
| Atwell T. Jarrett, | *Virginia.* |
| George Wilfred LeDuke, | *Massachusetts.* |
| Charles T. Lindsey, | *Virginia.* |
| Frank B. Maphis, | *Virginia.* |
| Wattie B. McGirt, | *South Carolina.* |
| Edwin J. Miller, | *Minnesota.* |
| Eli H. Neiman, | *Pennsylvania.* |
| A. Lee Penuel, | *Maryland.* |
| Walter W. Rowe, | *Pennsylvania.* |

| | |
|---|---|
| Hippolyte C. Salles, | *Louisiana.* |
| Carlos N. Sanchez, | *Cuba.* |
| Julian C. Smith, | *South Carolina.* |
| Walton O. Smith, | *Virginia.* |
| Myron W. Snyder, | *New York.* |
| Walter Stuart, | *Kentucky.* |
| Newton Addison Teague, | *South Carolina.* |
| Norman B. Tipton, | *Louisiana.* |
| J. Everett Toombs, | *Massachusetts.* |
| George Andreas Volck, | *Maryland.* |
| Fredrick Allen Weaver, | *Massachusetts.* |
| August F. L. Wietfeldt, | *Germany.* |

All the graduates, without a single exception, had attended *a full course* in the Dental Department.

Professor Gorgas announced the prizes in the dental department. The University Prize, a gold medal for the highest number of votes at the final examinations was awarded to John F. Garrett, of North Carolina; J. Edwin Miller, of Michigan receiving honorable mention. The S. S. White Prize, a dental engine, was awarded to F. Austin Banks, of Michigan; Frank G. Conklin, of New York, and J. E. Toombs, of Massachusetts, receiving honorable mention. George A. Volck, of Maryland, received the Snowden & Cowman Prize, a set of forceps. Myron W. Snyder, of New York, was awarded the Wilkerson Prize. W. O. Smith, of Virginia, receiving honorable mention. Eli H. Neiman, of Pennsylvania, received the Genese Prize, a set of instruments,. The "*Dental Register*" Prize was awarded to F. G. Conklin, of New York, and the "*Southern Dental Journal*" Prize, to Walter W. Rowe, of Pennsylvania.

Hon. John V. L. Findlay, Congressman elect, delivered the address to the graduates, in which he spoke of the great importance of the profession they had chosen, emphasizing the fact that there was yet much to be learned by the careful student of the ills which man is heir to, etc. He also called particular attention to the present age of skepticism, and the fact that the physician who was a thorough believer in the immortality of the human soul would necessarily be far more conscientious than he who believed that man was simply a piece of animated clay. He concluded as follows: "May yours be a career of honor, and may all the gold that glistens in your pathway, not be in the teeth of your patients, but may you receive for every plug a *quid pro quo.*"

